# *Streptococcus sinensis* Endocarditis outside Hong Kong

**DOI:** 10.3201/eid1308.080124

**Published:** 2007-08

**Authors:** Ilker Uçkay, Peter Rohner, Ignacio Bolivar, Béatrice Ninet, Marina Djordjevic, Vandack Nobre, Christian Garzoni, Jacques Schrenzel

**Affiliations:** * University Hospitals of Geneva, Geneva, Switzerland; † Institut für Angewandte Immunologie, Zuchwil, Switzerland

**Keywords:** Endocarditis, Streptococcus sinensis, dispatch

## Abstract

*Streptococcus sinensis* has been described as a causative organism for infective endocarditis in 3 Chinese patients from Hong Kong. We describe a closely related strain in an Italian patient with chronic rheumatic heart disease. The case illustrates that *S. sinensis* is a worldwide emerging pathogen.

Among the hundreds of bacteria that are pathogenic for humans, some are reposted only once and remain a rarity, while others are considered as emerging pathogens after several cases have been published. In 2002, Woo et al. from Hong Kong Special Administrative Region, People’s Republic of China, reported a new pathogen isolated from a 42-year-old Chinese woman with mitral regurgitation due to chronic rheumatic heart disease and infective endocarditis (HKU4) (*1*). Its 16S rRNA sequence (*2*) showed a new streptococcal species, subsequently named *Streptococcus sinensis* in honor of China; the sequencing showed that it was closely related to *Streptococcus gordonii* (96.4% homology) and to *Streptococcus intermedius* (96.3%) (*1*). Phenotypically, the species most closely resembled *S. intermedius*; some evidence suggests that *S. sinensis* could be the common ancestor of *S. anginosus* and *S. mitis* (*3*). In 2004, the same group published a retrospective analysis of 302 bacteremia cases caused by *S. viridans* in Hong Kong, including 2 other cases of endocarditis caused by *S. sinensis* with Lancefield group F (*4*). We describe another case of an infective endocarditis (or infection in general) due to *S. sinensis* outside Hong Kong.

## The Case

In December 1998, a 57-year-old Italian man with a severe mitral insufficiency of rheumatic origin underwent a nonbleeding dental procedure without antimicrobial prophylaxis. One month later, he was hospitalized at the University Hospitals of Geneva with fever (38°C–39°C), rigors, and weight loss of 4 kg in 3 weeks. There was no history of prior endocarditis, drug abuse, traumatism, or concomitant disease. At admission he was hemodynamically compensated and without fever. Over the cardiac apex, a grade 5/6 proto-mesosystolic murmur was audible. Transesophageal echocardiography showed vegetation on the mitral valve without abscesses. An ophthalmologic examination showed an embolus near the right macula. The urinary sediment exhibited microhematuria. In all blood-culture bottles, a gram-positive *Streptococcus* sp. was grown. Thus, infective endocarditis was diagnosed by the presence of 2 major and 3 minor criteria according to modified Duke criteria (*5*).

An antimicrobial drug treatment with intravenous (i.v.) penicillin G 6 × 4 million units/day and gentamicin 3× 1 mg/kg/day for 3 weeks was initiated. After an excellent clinical course and normalization of inflammation markers, treatment was switched to i.v. ceftriaxone 1× 2 g/day for another 3 weeks to enable outpatient therapy (*6*). No secondary abscesses occurred. Several infected teeth were extracted during treatment. Because of the severity of the preexisting mitral regurgitation, an elective replacement with a mechanical prosthesis was performed in March 1999. The patient was considered cured. Three years later he died of cerebral hematoma attributed to oral anticoagulation. Autopsy did not show any sequelae of former infection.

All 6 blood-culture bottles (3 BACTEC aerobic plus/F and 3 BACTEC anaerobic lytic/F; Becton Dickinson, Sparks, MD, USA.) were positive with gram-positive cocci in chains. The isolate grew as transparent α-hemolytic colonies (0.5- to 1-mm diameter) on sheep blood Columbia agar after an incubation of 24 h at 35°C in 4% CO_2_-enriched atmosphere. The biochemical identification system API 20 Strep (bioMérieux, Lyon, France) was used to attempt the identification. The numerical profile was 4241450, indicating *S. sanguinis* (95% similarity). Agglutination with Lancefield antisera (Streptokit, bioMérieux) was negative for groups A, B, C, D, F, and G. Antimicrobial MICs were determined with Etest (AB Biodisk, Solna, Sweden). Results were interpreted according the available Clinical Laboratory Standard Institute (CLSI, formerly NCCLS) criteria (*7*) (Table).

**Table:** Susceptibility of *Streptococcus sinensis*–related strain from University Hospitals of Geneva

Antimicrobial agent	MIC (mg/L)	Interpretation
Penicillin	0.064	Susceptible
Ceftriaxone	0.19	Susceptible
Imipenem	0.064	Susceptible
Gentamicin	3	Intermediate resistant
Levofloxacin	1	Susceptible
Clindamycin	0.094	Susceptible
Erythromycin	0.047	Susceptible
Rifampicin	0.032	Susceptible
Cotrimoxazole	1	Susceptible
Tetracycline	0.125	Susceptible
Tigecycline	0.064	Susceptible
Linezolid	0.5	Susceptible
Vancomycin	1	Susceptible
Teicoplanin	0.25	Susceptible

The genetic sequence of the 16S rRNA was determined by a capillary sequence analyzer (ABI 3130 XL DNA Analyzer, Applied Biosystems (Foster City, CA, USA) and compared with the nucleotide sequences in GenBank. Of >1,000 bp in the 16S rRNA sequence, only 2 differed from the previously published *S. sinensis* HKU4 (AF432856) (*1*), a sequence identical to that of HKU5 (AF432855) and HKU6 (AF432857) (*4*). Results were positive for identifying the superoxid dismutase (*sodA)* (primer from reference *8*) and RNA polymerase β-subunit (*rpoB)* (*9*) housekeeping gene sequences. Concerning *sodA*, for >404 bp there was 97.3% homology with the positive *S. sinensis* strain AY386220 (*9*) and only 2 bp differences in >286 bp (99.3% homology) with the *S. sinensis* strain EF451825. Concerning *rpoB***,** 485 bp were identical over 516 nt sequences (94% homology) of the *S. sinensis* strain AF199923 (*9*).

## Conclusions

We report a case outside Hong Kong of infective endocarditis caused by a strain of *S. sinensis*. Information on this novel pathogen has been published for 3 Chinese patients (*2*–*4*). Ours is the fourth published case worldwide. Because the original *S. sinensis* (*1*) is most closely related to our strain, we believe that our isolate belongs to that species. The close relationship of the sequence of our strain to the referential *sodA* and *rpoB* gene sequences also identifies our Geneva strain as *S. sinensis*. We have submitted the *sodA* and *rpoB* sequencing results to GenBank (accession nos. EF585234 and EF591041, respectively).

A 16S rRNA sequence with only 3 base differences from HKU4 had been detected by a German group in the aortic valve of another patient (GenBank accession no. AY049738, unpub. data), and other sequences have been reported by a French group (EF371928, unpub. data) and a British group (AY386220, unpub. data). We do not know more about these cases. Our Geneva strain differed only in 2 nt bases from the HKU4 strain (on positions 43 and 48) and only in 1 base (position 66) from the German sequence, whereas it is identical with the French sequence. This might suggest the emergence of a European strain of *S. sinensis*. From a clinical point of view, all reported patients (*1*,*4*) had an underlying chronic rheumatic heart disease of the mitral valve as the major risk factor. Only l patient had a preceding tooth extraction (*4*), like our patient with teeth abscesses. No other infections besides endocarditis caused by *S. sinensis* have been described thus far in humans or in animals. All previously reported patients were successfully treated by i.v. penicillin G or ampicillin for 4 weeks (combined therapy with gentamicin during the first 2 weeks) without necessity for surgical intervention. In light of other streptococcal endocarditic recommendations (*10*,*11*) and reported experiences with *S. sinensis* (*4*), our 6 weeks of antimicrobial drug treatment was probably excessive. The elective mitral valve replacement was indicated because of the severity of the preexisting regurgitation.

In conclusion, our case outside Hong Kong confirms that *S. sinensis* causes endocarditis throughout the world. Like other viridans streptococci, *S. sinensis* might be part of the human oral flora. The real number of cases is probably underestimated because commercial kits misidentify *S. sinensis* as *S. intermedius* or *S. anginosus* (*1*). With adequate sequencing technology, further reports may indicate the real prevalence of this emerging pathogen.
